# Subnormal vitamin B12 concentrations and anaemia in older people: a systematic review

**DOI:** 10.1186/1471-2318-10-42

**Published:** 2010-06-23

**Authors:** Wendy PJ den Elzen, Gerda M van der Weele, Jacobijn Gussekloo, Rudi GJ Westendorp, Willem JJ Assendelft

**Affiliations:** 1 Department of Public Health and Primary Care, Leiden University Medical Center, Leiden, The Netherlands; 2 Department of Gerontology and Geriatrics, Leiden University Medical Center, Leiden, The Netherlands

## Abstract

**Background:**

Pernicious anaemia is undeniably associated with vitamin B12 deficiency, but the association between subnormal vitamin B12 concentrations and anaemia in older people is unclear. The aim of this systematic review was to evaluate the association between subnormal vitamin B12 concentrations and anaemia in older people.

**Methods:**

Clinical queries for aetiology and treatment in bibliographic databases (PubMed [01/1949-10/2009]; EMBASE [01/1980-10/2009]) were used. Reference lists were checked for additional relevant studies. Observational studies (≥50 participants) and randomized placebo-controlled intervention trials (RCTs) were considered.

**Results:**

25 studies met the inclusion criteria. Twenty-one observational cross-sectional studies (total number of participants n = 16185) showed inconsistent results. In one longitudinal observational study, low vitamin B12 concentrations were not associated with an increased risk of anaemia (total n = 423). The 3 RCTs (total n = 210) were well-designed and showed no effect of vitamin B12 supplementation on haemoglobin concentrations during follow-up in subjects with subnormal vitamin B12 concentrations at the start of the study. Due to large clinical and methodological heterogeneity, statistical pooling of data was not performed.

**Conclusions:**

Evidence of a positive association between a subnormal serum vitamin B12 concentration and anaemia in older people is limited and inconclusive. Further well-designed studies are needed to determine whether subnormal vitamin B12 is a risk factor for anaemia in older people.

## Background

Pernicious anaemia is a form of anaemia that is undeniably associated with vitamin B12 deficiency. Finding the cure for pernicious anaemia even led to the discovery of vitamin B12 [1-7]. Nowadays, vitamin B12 deficiency is not only associated with (pernicious) anaemia, but is also linked with other conditions such as dementia, neuropathy and subacute combined degeneration of the spinal cord [8-11]. Therefore, individuals with low serum concentrations of vitamin B12 are frequently given vitamin B12 supplements [10-12]. Also, since low serum vitamin B12 concentrations are very common in older individuals [13], screening older people for vitamin B12 deficiency has often been recommended [14,15].

Although the biological role of vitamin B12 in haematopoiesis is well-defined [11,16-18], the outcomes of some studies cast doubt on the relationship between low vitamin B12 concentrations and anaemia in older individuals [19,20]. The association between low vitamin B12 and anaemia has become even more difficult to study because various cut-off values for serum vitamin B12 are used and serum concentrations of vitamin B12 appear not to be an accurate reflection of vitamin B12 status at the tissue level [8,21,22].

Therefore, in order to evaluate the association between subnormal vitamin B12 concentrations and anaemia in older subjects, we performed a systematic review of relevant observational (cross-sectional and longitudinal) studies and randomized placebo-controlled trials that have been published in the medical literature to date.

## Methods

### Criteria for considering studies for this review

All published cross-sectional and longitudinal observational studies in older individuals (mean or median age ≥60 years) on the association between vitamin B12 and anaemia were considered for inclusion in this review. The exact definitions of vitamin B12 deficiency and anaemia had to be clearly stated. Observational studies with less than 50 participants were excluded.

In addition, we considered all randomized controlled trials (RCTs) where subjects 60 years of age and over were treated with vitamin B12 (any dose and any form of administration) and were compared with subjects who were given a placebo. Studies in which patients had received vitamin B12 treatment prior to the study were excluded. Trials in which combinations of vitamin B12 and folic acid were administered were also excluded.

### Search strategy

We used predefined clinical queries (both sensitive and specific) for aetiology and treatment as provided in PubMed (January 1949 - October 2009) and EMBASE (January 1980 - October 2009) using relevant MeSH-headings and free text words for vitamin B12 and anaemia. Case reports and letters were excluded. We restricted our search to articles published in English, French, German and Dutch. The exact search strategies are presented in Additional file 1 (PubMed) and Additional file 2 (EMBASE). Articles in which the mean or median age of the study population was less than 60 years were excluded by hand during the selection process.

### Selection of studies

All titles and abstracts retrieved from the electronic databases were initially assessed by the first author. In cases of uncertainty of relevance, the titles and abstracts were also independently assessed by the second author. Disagreement was resolved by consensus. Full copies were obtained for papers that were potentially relevant to our review. Both authors independently assessed these papers and disagreement was again resolved by consensus. Furthermore, reference lists given in the full papers were scrutinized by the first author for additional relevant studies.

### Data extraction

From the papers describing observational studies, (i) mean/median (SD) vitamin B12 concentrations in anaemic and non-anaemic older subjects, (ii) mean/median (SD) haemoglobin concentrations in older subjects with vitamin B12 deficiency and subjects with normal vitamin B12 concentrations, (iii) percentage of subjects with vitamin B12 deficiency in anaemic and non-anaemic older subjects, (iv) percentage of subjects with anaemia in vitamin B12 deficient and non-deficient older subjects, or (v) the correlation between vitamin B12 concentrations and haemoglobin concentrations were extracted. Data describing the correlation between vitamin B12 concentrations and mean corpuscular volume (MCV) or macrocytosis were also extracted, if available. From the papers describing intervention studies, we extracted pre- and post-treatment mean haemoglobin concentrations, mean proportions of haematocrit and mean MCV, or the change in these variables during treatment. Measures of association between low vitamin B12 concentrations and anaemia and mean corpuscular volume (MCV), and indicators of statistical significance were also extracted.

### Quality assessment and level of evidence

The first and second author independently assessed the quality of every included study. The included longitudinal study that had been performed by our own research group was also independently assessed by an independent investigator (DAWM van der Windt). Disagreement was resolved by consensus.

The methodological quality assessment of the observational studies was based on previously developed checklists for such studies [23,24]. For the cross-sectional observational studies, we only used items on the checklists relevant to the quality assessment of cross-sectional studies, including the use of valid selection criteria, a response of ≥80%, the use of a valid and reproducible method to assess the exposure, the use of a valid and reproducible method to assess the outcome, the use of new and incident patients, adjustment for possible confounders, and the inclusion of more than 100 subjects.

One point was awarded for each question that was answered by 'yes'. The answers 'no' or 'unknown' were given 0 points. The maximum total score possible for cross-sectional studies was 7 points. Cross-sectional studies that scored 5 points or more in terms of their quality assessment were considered 'high quality'.

For the longitudinal studies, we added two items to the quality assessment, namely a response at main moment of follow-up ≥80%, and data collection for ≥1 year.

Again, one point was awarded for each question that was answered by 'yes' and the answers 'no' or 'unknown' were given 0 points. The maximum total score possible for longitudinal studies was 9 points. Longitudinal studies that scored 7 points or more in their quality assessment were considered 'high quality'.

The quality assessment of the intervention studies was carried out according to the criteria developed by Jadad and colleagues [25]. This checklist includes three main questions on the reporting of randomization, blinding, and withdrawals and dropouts.

For each question that was answered by 'yes', one point was awarded. An additional point was given if the method used to generate the sequence of randomization was described and was appropriate or if the method of double blinding was described and was appropriate. One point was deducted if the method to generate the sequence of randomization was described and it was inappropriate or if the method of double blinding was described and it was inappropriate. The maximum total score possible was 5 points. Intervention studies which scored 3 points or more for their quality assessment were considered 'high quality'.

The level of evidence for the association between a subnormal vitamin B12 and anaemia was graded according to the criteria described by the GRADE working group, separately for the observational studies and the intervention studies [26].

## Results

### Selection of studies

**Figure 1:**
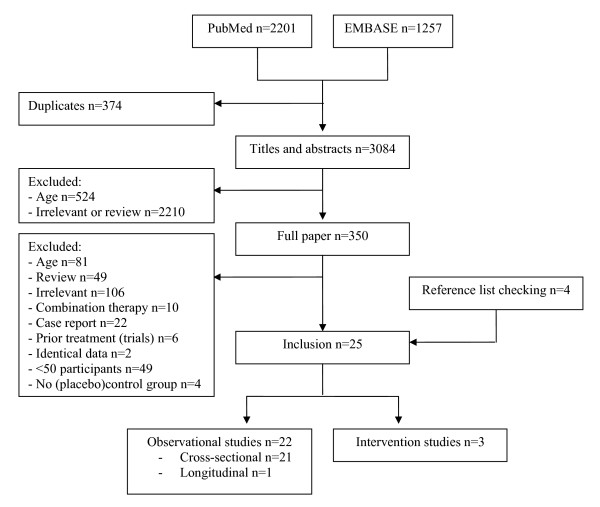
**Schematic representation of the selection of publications for review**.

Electronic searches of PubMed and EMBASE databases identified 3084 titles and abstracts of papers relevant to the present review. We obtained 350 full papers; 21 of which met our inclusion criteria. Four additional papers were obtained by examining the reference lists given in these chosen papers. A schematic representation of the search process is described in Figure 1.

### Observational studies

Twenty-one cross-sectional observational studies with a total number of 16185 participants were included (Table 1) [27-47]. Eleven studies included participants from the general population [27-37]. Ten studies investigated the association between vitamin B12 and anaemia in hospitalized or institutionalized subjects [38-47]. Detailed information about the studies can be found in Additional file 3.

**Table 1:** Summary of the results and quality assessment of the observational studies included in this review

Author	Year of publication	Sample size (N)	Age of subjects (years)	Study population	Presence of an association between subnormal vitamin B12 and anaemia	Quality of study*
*Cross-sectional*						

Population-based						

Allain [27]	1997	233	≥60, median 72	Random sample of older Zimbabweans (rural and urban).	?	5

Björkegren [28]	2001	224	≥70, mean 78.0 (95% CI 77.2-78.9)	Random sample of persons aged 70 years and over in the Borough of Älvkarleby in the county of Uppsala, Sweden	-	6

Clarke [29]	2008	2257	≥65, mean 79.2 (SD 6.2)	Oxford Healthy Aging Project: Random sample from general practice registers of people ≥65 years living in Oxford, UK. Banbury B12 Study: Random sample of people aged ≥75 years living in their own homes and registered with general practices in Banbury, Oxfordshire, UK.	+	6

Hin [30]	2006	1000	≥75, mean 81.4 (SD 4.6)	Random sample of people ≥75 years living at home, registered with general practitioners in Banbury, England	-	4

Hvas [31]	2005	937	Median 72, range 19-102	Subjects with increased MMA (>0.28 μmol/L) within in Aarhus, Denmark, from 1995-2000.	-	5

Johnson [32]	2003	103	≥60, mean 76.4 (SD 8.1)	Older individuals enlisted in nutrition service program in rural northeast Georgia, USA.	?	4

Lippi[33]	2009	878	Range 85-101	Unselected subjects older than 85 years, who were referred by general practitioners to a laboratory in Verona, Italy, for routine diagnostic check-up over a period of 2 years	-	6

Loikas [34]	2007	1048	≥65, 37% ≥75	Lieto study; unselected population based health survey in Lieto, Finland.	-	6

McLennan [35]	1973	347	>65	Random sample of people >65 years living at home in Kilsyth and Northern Glasgow, UK.	?	5

Morris [36]	2007	1459	≥60, mean 70 (SEM 0.32)	Non-institutionalized civilian population (NHANES), USA.	+	5

Penninx [37]	2000	700	≥65, mean 77.3	Physically disabled older women living in the community (Women's Health and Ageing Study), Baltimore area, USA	?	4

Hospitalized/Institutionalized						

Bisbe[38]	2009	599	Mean 68 (SD 13)	All consecutive patients scheduled for major orthopaedic surgery for which blood was routinely grouped preoperatively in University Hospital in Barcelona, Spain.	-	5

Chui [39]	2001	3453	48% >70	All patients admitted to the Prince of Wales hospital, Hong Kong, with vitamin B12 measurements in 1996.	-	5

Joosten [40]	1990	292	>65	Consecutive patients admitted to the geriatric department of the University Hospital, Leuven, Belgium	?	5

Kwok [41]	2002	96	>55, mean age >78.0	Female ambulatory vegetarians (>3 years) in Hong Kong.	?	5

Metz [42]	1996	94	Cases: mean 79.8 Controls: mean 80.7	Patients with suspected low vitamin B12 levels based on clinical examination by attending staff in Royal Melbourne and North West hospitals, Australia. If low vitamin B12: case. If normal vitamin B12: control.	-	2

Mooney [43]	2004	905	65-85	Hospitalized patients in Belfast, Ireland, who had vitamin B12, folate, Hb, MCV and ferritin measured within ±4 days of each other in February-July 2003	-	3

Prayurahong [44]	1993	147	≥60	Subjects visiting clinic for older individuals in Rajvithi Hospital, Bangkok	?	3

Stott [45]	1997	290	Range 62-110	Consecutive new referrals to a geriatric medical unit in Glasgow, Scotland.	-	5

Wang [46]	2009	827	Mean 77.1 (SD 7.5), range 60-96	Patients in the department of Neurology of Shanghai Punan Hospital, Shanghai, China.	+	4

Witte [47]	2004	296	Mean 72.5 (10.3)	Consecutive patients with chronic heart failure attending a heart failure clinic in Cottingham, UK	-	4

*Longitudinal*						

Den Elzen [48]	2008	423	85	All 85-year-old inhabitants of Leiden, the Netherlands. Participants using vitamin B12 or folate supplements at baseline or during follow-up were excluded.	-	7

*Based on checklists from van der Windt et al [23,24]. Higher scores indicate higher quality (range cross-sectional studies 0-7; longitudinal studies 0-9). Cross-sectional studies that scored 5 points or more were considered 'high quality'. Longitudinal studies that scored 7 points or more were considered 'high quality'.

We did not try to retrieve a pooled estimate of the results of the cross-sectional studies for the following reasons: 1) the studies had been performed in very different patient populations, 2) the investigators had used different cut-off points for vitamin B12 deficiency and anaemia, 3) different effect estimates had been calculated and 4) the overall methodological quality was poor. This clinical and methodological heterogeneity could not be solved by any subgroup analysis.

Of the 21 observational cross-sectional studies, 8 studies were of low quality ("see Additional file 4") [30,32,37,42-44,46,47]. The remaining 13 studies of high quality differed substantially in sample size, criteria for low vitamin B12 status and level of adjustment for confounders [27-29,31,33-36,38-41,45]. The studies showed inconsistent results with regards to the association between subnormal vitamin B12 concentrations or vitamin B12 deficiency and anaemia in older subjects. In three studies, an association between subnormal vitamin B12 and anaemia was found (Clarke et al [29], Morris et al. [36] and Wang et al [46]). For seven studies, the presence of an association was not clear because conflicting findings regarding the presence of an association were reported (Allain et al. [27], Johnson et al. [32], McLennan et al. [35], Penninx et al. [37], Joosten et al. [40], Kwok et al. [41], and Prayaharong et al. [44]). Eleven studies did not find an association between subnormal vitamin B12 and anaemia (Bjorkegren et al. [28], Hin et al. [30], Hvas et al. [31], Lippi et al. [33], Loikas et al. [34], Bisbe et al. [38], Chui et al. [39], Metz et al. [42], Mooney et al. [43], Stott et al. [45], and Witte et al. [47]).

Allain et al., McLennan et al. and Joosten et al. had applied the lowest serum vitamin B12 cut-off points for vitamin B12 deficiency (100 pg/mL, 140 pg/mL and 110 pmol/L respectively) [27,35,40]. In these three studies, the presence of an association between vitamin B12 deficiency and anaemia was not clear, because conflicting findings regarding the presence of an association were reported [27,35,40]. In the two largest population-based studies by Clarke et al. and Morrison et al., subjects with low vitamin B12 concentrations had an increased risk of having anaemia, also after extensive adjustment for confounders [29,36], but an even larger study in hospitalized older persons by Chui et al. did not show any association between vitamin B12 deficiency and anaemia [39]. Similar inconsistencies were found with respect to the association between subnormal vitamin B12 concentrations and MCV.

Our own study appeared to be the only longitudinal study on the effect of low vitamin B12 concentrations (<150 pmol/L) on developing anaemia in a population-based sample of 85-year-old subjects (n = 423) [48]. Detailed information about the study and the quality assessment can be found in Additional file 5 and Additional file 6, respectively. After adjustment for possible confounding variables, low vitamin B12 concentrations were not associated with an increased risk of having anaemia at baseline (prevalent anaemia) or developing anaemia during follow-up (incident anaemia, Table 1).

### Intervention studies

We found three randomized placebo-controlled trials with a total number of 210 participants that met the inclusion criteria for intervention studies for our review (Table 2) [49-51]. Detailed information about the trials can be found in Additional file 7. These three trials included patients with low or subnormal vitamin B12 levels concentrations at the start of the study. The first trial by Hughes and colleagues included a random sample of 39 persons aged ≥65 years registered at general practices in a town in Wales, UK, that were treated for 4 weeks with intramuscular hydroxocobalamin or placebo. Haemoglobin was measured after 5 weeks [49]. The second trial by Hvas et al. included 140 persons in Aarhus, Denmark, with elevated methylmalonic acid levels (median age 75 years in the treatment group and 74 years in the placebo group) that received weekly intramuscular injections of cyanocobalamin of placebo for 1 month. Haemoglobin was measured after 3 months (13 weeks) [50]. In the third trial by Seal and colleagues, 31 persons in two geriatric hospitals in Melbourne, Australia, (mean age ≥78 years) received two different doses of oral cyanocobalamin daily or placebo for 4 weeks [51].

**Table 2:** Summary of the results and quality assessment of the intervention studies included in this review

Author	Year of publication	Sample size (N)	Age of subjects (years)	Study population	Intervention	Effectiveness of vitamin B12 treatment on haemoglobin concentrations	Quality of study*
Hughes [49]	1970	Placebo n = 19, Treatment n = 20	≥ 65	Random sample of subjects aged ≥65 years from general practices in a town in Wales, UK. Subjects with vitamin B12 level <150 pg/mL were invited to participate in the trial. None had anaemia or evidence of vitamin B12 neuropathy and none was taking drugs that might interfere with vitamin B12 assays or anticonvulsants that might reduce the serum vitamin B12 level.	Intramuscular hydroxocobalamin (1000 μg), twice in the first week, and then at weekly intervals for a further four weeks or placebo containing a matching solution of phenol red (phenylsulphonphtalein 0.075%)	-	4 points

Hvas [50]	2001	Placebo n = 70, treatment n = 70	Treatment group: Median 75 (range 19-92) Placebo group: 74 (range 33-88)	140 subjects in Aarhus, Denmark, with elevated methylmalonic acid (P-MMA, 0.40-2.00 μmol/L) which had not received prior vitamin B12 treatment. Participants were enrolled between November 1998 and March 2000. Exclusion criteria: low Hb levels, low ferritin levels, TSH≥4.1 mIU/L, high creatinine levels, life-threatening disease, treatment with anticoagulants, tropical atoxic neuropathy.	Weekly intramuscular injections of 1 mg cyanocobalamin (n = 70) or placebo containing 1 mL of isotonic sodium chloride (n = 70) for 1 month	-	4 points

Seal [51]	2002	Placebo n = 11, oral vitamin B12 10 μg n = 10, oral vitamin B12 50 μg, n = 10	Mean age placebo 78, vitamin B12 10 μg 82, vitamin B12 50 μg 85	31 patients in two geriatric hospitals in Melbourne, Australia with subnormal serum vitamin B12 (100-150 pmol/L) discovered as part of their clinical assessment. Exclusion criteria: known neoplasm, life-threatening or terminal illness, history of malabsorption, pernicious anaemia, anaemia of other cause, prior vitamin B12 treatment or vitamin supplementation, neurological disorder other than stroke.	Placebo (Australian Pharmaceutical Formulary (APF) red mixture and APF hydrobenzoate compound), 10 μg oral cyanocobalamin or 50 μg oral cyanocobalamin daily for 4 weeks	-	2 points

**Based on checklist from Jadad et al. [25] Higher scores indicate higher quality (range 0-5). Intervention studies that scored 3 points or more were considered 'high quality'.

Due to clinical heterogeneity (differences in methods of administration, dose of vitamin B12, outcome measures and treatment follow-up time) we did not combine the results in a meta-analysis. However, all three RCTs, of which two were regarded high quality ("see Additional file 8") [49,50], showed no beneficial effect of vitamin B12 administration on haemoglobin concentrations and MCV. In the study by Hughes et al, there was, on average, a small decrease in haemoglobin level during the trial but the difference between the mean changes in those given vitamin B12 and those given placebo was very small and not statistically significant [49]. In the study by Hvas et al, the change in haemoglobin level was the same in the vitamin B12 group and placebo group [50]. In addition, no differences were observed in the mean change in haemoglobin level in the three treatment groups in the study by Seal et al. [51].

### Level of evidence

Because of serious limitations of study quality and important inconsistency of the observational studies, the level of evidence for an association between subnormal vitamin B12 concentrations and anaemia in older individuals was considered low. Since the quality of two of the RCTs was considered high, the level of evidence for the lack of effect of vitamin B12 administration on haemoglobin in older persons with low vitamin B12 concentrations at the start of the study was considered high.

## Discussion

Although anaemia is regarded as a classic complication of vitamin B12 deficiency in older people [8,9,11], this systematic review showed that evidence for an association between a subnormal serum vitamin B12 concentration and anaemia in older people is limited and inconclusive. A total of 21 observational cross-sectional studies included in our review showed inconsistent results [27-47]. Similar inconsistencies were found with respect to the association between subnormal vitamin B12 concentrations and MCV. We identified one longitudinal observational study in older subjects in the general population, in which low vitamin B12 concentrations were not associated with an increased risk of developing anaemia or the change in MCV over time [48]. Interestingly, the three included RCTs, of which two were regarded as high quality RCTs, showed no effect of vitamin B12 administration on haemoglobin concentrations and MCV in subjects with subnormal vitamin B12 concentrations at the start of the study [49-51], which is unlikely to be explained by insufficient dose of vitamin B12 supplementation, since the RCTs did show changes in vitamin B12, homocysteine and MMA concentrations.

This lack of effect of vitamin B12 treatment on haemoglobin concentrations and MCV in patients with low or subnormal vitamin B12 concentrations is surprising, especially since vitamin B12 has such a well-defined role in haematopoiesis [11,16-18]. However, our results are in line with a systematic review by Oosterhuis and co-workers that showed that the diagnostic value of the mean corpuscular volume in the detection of vitamin B12 deficiency is poor [52]. There are two possible explanations why evidence of an association between subnormal vitamin B12 concentrations and anaemia is lacking. First, the studies included in the review had used many different tests to measure the concentration of serum vitamin B12, ranging from microbiological assays with L. leichmannii to radio immunoassays, and had applied different cut-off points for vitamin B12 deficiency. This may have led to misclassification in the diagnosis of vitamin B12 deficiency and, as a result, to dilution of the association between vitamin B12 deficiency and anaemia. However, the 3 studies that had applied the lowest cut-off point for vitamin B12 deficiency, in which therefore the strongest associations were to be expected, did not report clear associations between vitamin B12 deficiency and anaemia [27,35,40]. The second explanation is that a subnormal vitamin B12 concentration alone is not a sufficient cause to develop anaemia. Other genetic or environmental factors may be necessary to develop anaemia in the presence of subnormal vitamin B12 concentrations. A similar explanation has been proposed for the lack of association between the C282Y mutation of the hereditary haemochromatosis gene and mortality in old age [53].

Several difficulties were encountered when conducting this systematic review. These are similar to the problems Oosterhuis and co-workers faced when writing their systematic review on the diagnostic accuracy of the mean corpuscular volume in the detection of vitamin B12 deficiency [52]. Most importantly, the relation between low vitamin B12 concentrations and anaemia was not an explicit research question in most of the studies included in the present review, which could have resulted in insufficient statistical power to detect an association.

The diagnosis of vitamin B12 deficiency is a major concern in medical literature and its difficulties have been addressed thoroughly by others in the field [9,54]. Our systematic literature search identified only 25 relevant studies to review the association between vitamin B12 and anaemia in older people. In those studies many different assays were used to measure vitamin B12 and haemoglobin concentrations and varying cut-off points were applied to define vitamin B12 deficiency and anaemia. This considerably limited the comparability of the studies and emphasizes the need for a clear and globally used definition of (sub)normal vitamin B12 concentrations, either based on serum vitamin B12 concentrations alone, or in combination with elevated homocysteine or methylmalonic acid concentrations [9].

Furthermore, the clinical and methodological heterogeneity in the included observational studies prevented us from performing statistical pooling of data and thus drawing definite conclusions. The participation rates and adjustments for potential confounders, in particular, warrant improvement in future observational studies. We identified only one longitudinal study on this topic. Although this longitudinal study did not show any association between low serum vitamin B12 concentrations and the future development of anaemia, this study involved subjects aged 85 years only and has to be replicated in younger age groups (60-85 years) before more definite conclusions can be drawn.

We found 3 placebo-controlled trials meeting our inclusion criteria. Participants in these trials received vitamin B12 supplements for 4 weeks and were only followed for 1 to 3 months. Randomized placebo-controlled trials with longer treatment and longer follow-up periods are needed, because the effects of vitamin B12 supplementation on haemoglobin concentrations may only become apparent after 3 months.

Although we did not find an association between subnormal vitamin B12 concentrations and anaemia in older people, this does not imply that patients with pernicious anaemia or age-related food-vitamin B12 malabsorption (with tissue depletion of vitamin B12 and very low vitamin B12 concentrations) will not benefit from vitamin B12 administration, especially since non-placebo-controlled studies showed (large) increases in haemoglobin concentrations or haematocrit after intramuscular or oral vitamin B12 administration in patients with pernicious anaemia or age-related food-vitamin B12 malabsorption [55-59]. However, apart from the undisputed reality of pernicious anaemia, the clinical impact of a subnormal vitamin B12 level in older people is unclear, especially since several observational studies and randomized controlled trials also showed no effect of vitamin B12 administration on cognitive function [60-62].

## Conclusions

The studies included in this systematic review indicate that evidence of an association between a subnormal serum vitamin B12 concentration and anaemia in older people is limited and inconclusive. If anything, given the high clinical relevance of our research question, we recommend more well-designed longitudinal observational studies, in younger age groups (60-85 years) especially, and intervention studies of appropriate size and duration with timely follow-up periods to determine whether a subnormal vitamin B12 is a risk factor for anaemia in older people.

## Competing interests

The authors declare that they have no competing interests.

## Authors' contributions

WPJdE was involved in the conception and design of the review, performed the systematic literature search, assessed all abstracts and full copies, assessed the quality of the included papers, was involved in the analysis and interpretation of the data, and drafted the first version of the manuscript. GMvdW was involved in the conception and design of the review, assessed abstracts and full copies, assessed the quality of the included papers, was involved in the analysis and interpretation of the data, and critically revised the manuscript for important intellectual content. JG was involved in the conception and design of the review, was involved in the interpretation of the data, and critically revised the manuscript for important intellectual content. RGJW was involved in the interpretation of the data and critically revised the manuscript for important intellectual content. WJJA was involved in the conception and design of the review, was involved in the analysis and interpretation of the data, and critically revised the manuscript for important intellectual content. All authors read and approved the final manuscript.

## Pre-publication history

The pre-publication history for this paper can be accessed here:

http://www.biomedcentral.com/1471-2318/10/42/prepub

## Supplementary Material

Additional file 1 Strategy used to search PubMed database for publications on subnormal vitamin B12 levels and anaemia (carried out October 2009) Click here for file

Additional file 2 Strategy used to search EMBASE database for publications on subnormal vitamin B12 levels and anaemia (carried out October 2009) Click here for file

Additional file 3 Observational cross-sectional studies on aetiology of vitamin B12 deficiency and anaemia in older subjects included in the present review Click here for file

Additional file 4 Quality assessment of cross-sectional observational studies on aetiology of vitamin B12 deficiency and anaemia in elderly subjects included in the present review Click here for file

Additional file 5 Observational longitudinal study on aetiology of vitamin B12 deficiency and anaemia in older subjects included in the present review Click here for file

Additional file 6 Quality assessment of longitudinal observational studies on aetiology of vitamin B12 deficiency and anaemia in elderly subjects included in the present review Click here for file

Additional file 7 Randomized placebo-controlled trials of the effect of vitamin B12 administration on haemoglobin levels in older subjects included in the present review Click here for file

Additional file 8 Quality assessment of randomized placebo-controlled trials on the effect of vitamin B12 administration on haemoglobin levels in elderly subjects included in the present review Click here for file
