# Supplementary material for: Subnormal vitamin B12 concentrations and anaemia in older people: a systematic review
Source: BMC Geriatr. 2010 Jun 23;10:42. doi: 10.1186/1471-2318-10-42 (PMC2900261; doi:10.1186/1471-2318-10-42)
Supplement: Additional file 1 — Strategy used to search PubMed database for publications on subnormal vitamin B12 levels and anaemia (carried out October 2009) [file 1471-2318-10-42-S1.DOC]

**Additional file 1** Strategy used to search PubMed database for publications on subnormal vitamin B12 concentrations and anaemia (carried out October 2009)

| #1 | Vitamin B 12[MeSH] |
| --- | --- |
| #2 | Vitamin B 12 deficiency[MeSH] |
| #3 | vitamin B12[tw] OR vitamin B 12[tw] OR cobalamin*[tw] OR cyanocobalamin*[tw] OR hydroxocobalamin*[tw] OR hydroxycobalamin*[tw] |
| #4 | #1 OR #2 OR #3 |
| #5 | Anemia[MeSH] |
| #6 | Hemoglobins[MeSH] |
| #7 | anemi*[tw] OR anaemi*[tw] OR hemoglobin*[tw] OR haemoglobin*[tw] |
| #8 | #5 OR #6 OR #7 |
| #9 | #4 AND #8 |
| #10 | (#9) AND (risk* [Title/Abstract] OR risk* [MeSH:noexp] OR risk *[MeSH:noexp] OR cohort studies[MeSH Terms] OR group*[Text Word]) |
| #11 | (#9) AND ((relative[Title/Abstract] AND risk*[Title/Abstract]) OR (relative risk[Text Word]) OR risks[Text Word] OR cohort studies[MeSH:noexp] OR (cohort[Title/Abstract] AND stud*[Title/Abstract])) |
| #12 | (#9) AND ((clinical[Title/Abstract] AND trial[Title/Abstract]) OR clinical trials[MeSH Terms] OR clinical trial[Publication Type] OR random*[Title/Abstract] OR random allocation[MeSH Terms] OR therapeutic use[MeSH Subheading]) |
| #13 | (#9) AND (randomized controlled trial[Publication Type] OR (randomized[Title/Abstract] AND controlled[Title/Abstract] AND trial[Title/Abstract])) |
| #14 | (#9) AND systematic[sb] |
| #15 | #10 OR #11 OR #12 OR #13 OR #14 |
| #16 | #15 NOT case reports NOT letter |
| #17 | #16 Limits: English, French, German, Dutch |
| Ad. 10 PubMed Clinical Queries; Etiology; Broad, sensitive search  Ad. 11 PubMed Clinical Queries; Etiology; Narrow, specific search  Ad. 12 PubMed Clinical Queries; Therapy; Broad, sensitive search  Ad. 13 PubMed Clinical Queries; Therapy; Narrow, specific search | |
