# Supplementary material for: Subnormal vitamin B12 concentrations and anaemia in older people: a systematic review
Source: BMC Geriatr. 2010 Jun 23;10:42. doi: 10.1186/1471-2318-10-42 (PMC2900261; doi:10.1186/1471-2318-10-42)
Supplement: Additional file 2 — Strategy used to search EMBASE database for publications on subnormal vitamin B12 levels and anaemia (carried out October 2009) [file 1471-2318-10-42-S2.DOC]

| 1 | cobalamin/ or cyanocobalamin/ or hydroxocobalamin/ |
| --- | --- |
| 2 | Cyanocobalamin Deficiency/ |
| 3 | (cobalamin$ or cyanocobalamin$ or hydroxocobalamin$ or hydroxycobalamin$).mp. [mp=title, abstract, subject headings, heading word, drug trade name, original title, device manufacturer, drug manufacturer name] |
| 4 | (vitamin B12 or vitamin B 12).mp. [mp=title, abstract, subject headings, heading word, drug trade name, original title, device manufacturer, drug manufacturer name] |
| 5 | 1 or 2 or 3 or 4 |
| 6 | exp Anemia/ |
| 7 | exp Hemoglobin/ |
| 8 | (anemi$ or anaemi$ or hemoglobin$ or haemoglobin$).mp. [mp=title, abstract, subject headings, heading word, drug trade name, original title, device manufacturer, drug manufacturer name] |
| 9 | 6 or 7 or 8 |
| 10 | 5 and 9 |
| 11 | limit 10 to (dutch or english or french or german) |
| 12 | Case Report/ |
| 13 | 11 not 12 |
| 14 | limit 13 to "causation-etiology (sensitivity)" |
| 15 | limit 13 to "causation-etiology (specificity)" |
| 16 | limit 13 to "treatment (2 or more terms high sensitivity)" |
| 17 | limit 13 to "treatment (2 or more terms high specificity)" |
| 18 | 14 or 15 or 16 or 17 |
| Ad. 14 risk:.mp. or exp methodology/ or exp epidemiology/  Ad. 15 (cohort or relative risk:).tw.  Ad. 16 random:.tw. or clinical trial:.mp. or exp health care quality/  Ad. 17 (double-blind: or placebo:).mp. or blind:.tw.  Reference: Health Information Research Unit. Search Strategies for EMBASE in ovid syntax. Accessed 25 April 2008. [http://hiru.mcmaster.ca/hedges/All-EMBASE.htm](https://mail.lumc.nl/exchweb/bin/redir.asp?URL=http://hiru.mcmaster.ca/hedges/All-EMBASE.htm) | |

**Additional file 2** Strategy used to search EMBASE database for publications on subnormal vitamin B12 concentrations and anaemia (carried out October 2009)
