# Supplementary material for: Subnormal vitamin B12 concentrations and anaemia in older people: a systematic review
Source: BMC Geriatr. 2010 Jun 23;10:42. doi: 10.1186/1471-2318-10-42 (PMC2900261; doi:10.1186/1471-2318-10-42)
Supplement: Additional file 3 — Observational cross-sectional studies on aetiology of vitamin B12 deficiency and anaemia in older subjects included in the present review [file 1471-2318-10-42-S3.DOC]

**Additional file 3** Observational cross-sectional studies on aetiology of vitamin B12 deficiency and anaemia in older subjects included in the present review

| Author | Year of publication | Sample size (N) | Age of subjects (years) | Study population | Vitamin B12 (VitB12): analysis and limits for deficiency | Haemoglobin (Hb): analysis and limits for anaemia | Presence of an association | Quality of study* |  |
| --- | --- | --- | --- | --- | --- | --- | --- | --- | --- |
| *Population-based* | | | | | | | | | |
| Allain [27] | 1997 | 233 | ≥60, median 72 | Random sample of older Zimbabweans (rural and urban), enrolled between October 1994 and March 1995. | Chemiluminescence method  Vitamin B12 deficiency (vitamin B12<100 pg/mL [74 pmol/L]): prevalence 13% | Method not specified. Anaemia (women Hb<12 g/dL and men Hb<13 g/dL): 23% | Unclear:  1) Vitamin B12 concentrations did not correlate with Hb.  2) Vitamin B12 significantly lower in those who were anaemic (290 vs 413 pg/ml, p<0.05).  3) No relationship between vitamin B12 and MCV. | 5 points |  |
| Björkegren [28] | 2001 | 224 | ≥70, mean 78.0 (95% CI 77.2-78.9) | Random sample of persons aged 70 years and over in the Borough of Älvkarleby in the county of Uppsala, Sweden, sampled in 1993 and 1995 | Competitive radioligand method | Method not specified. | No:  Multivariate regression correlation coefficient vitamin B12 for Hb 0.0004 (95% CI -0.007; 0.008) | 6 points |  |
| Clarke [29] | 2008 | 2257 | ≥65, mean 79.2 years (SD 6.2) | Oxford Healthy Aging Project: Random sample from general practice registers for people ≥65 years living in Oxford, UK, sampled in 1995.  Banbury B12 Study:  Random sample of people aged ≥75 years living in their own homes and registered with general practices in Banbury, Oxfordshire, UK, enrolled between March 2003 and April 2004. | Chemiluminescence system. Study population divided in 3 groups based on tertiles of vitamin B12. Mean vitamin B12 in lowest tertile 167 pmol/L | Method not specified. Anaemia (women <110 g/L and men <120 g/L): 7%. | Yes:  Participants in lowest tertile of vitamin B12 had increased risk of anaemia compared to participants in the highest tertile (adjusted OR 1.32 (95% CI 1.03-1.70) | 6 points |  |
| *Based on checklists from van der Windt et al [23,24] | | | | | | | | | |

**Additional file 3** continued

| Author | Year of publication | Sample size (N) | Age of subjects (years) | Study population | Vitamin B12 (VitB12): analysis and limits for deficiency | Haemoglobin (Hb): analysis and limits for anaemia | Presence of an association | Quality of study* |  |
| --- | --- | --- | --- | --- | --- | --- | --- | --- | --- |
| Hin [30] | 2006 | 1000 | ≥75 s, mean 81.4 (SD 4.6) | Random sample of people ≥75 years living at home, registered with general practitioners in Banbury, England, enrolled between March 2003 and April 2004. | Immunoassay. Low serum vitamin B12 (<133 pmol/L): n=125, normal serum vitamin B12 levels n=875. | Standard methods (not specified). | No:  1) Mean (SD) Hb for normal vitamin B12 group 13.2 (1.4) g/dL and for low vitamin B12 group 13.2 (1.2) g/dL, P>0.05  2) Mean (SD) MCV for normal vitamin B12 group 91.3 (5.0) fL and for low vitamin B12 group 92.8 (5.4) fL, P=0.0025 | 4 points |  |
| Hvas [31] | 2005 | 937 | Median 72, range 19-102 | Subjects with increased MMA (>0.28 µmol/L) within in Aarhus, Denmark, from 1995-2000. | Chemiluminescence method by a competitive protein binding assay. Low vitamin B12 levels: <200 pmol/L | Method not specified. Low Hb levels: <7.4 mmol/L for women and <8.4 mmol/L for men. Prevalence of anaemia: 10% | No:  1) Symptoms of anaemia were not associated with vitamin B12 deficiency: adjusted OR 1.04 95% CI 0.67-1.61  2) 27 individuals with macrocytic anaemia did not have lower vitamin B12 than individuals who did not have macrocytic anaemia. | 5 points |  |
| Johnson [32] | 2003 | 103 | ≥60, mean 76.4 (SD 8.1) | Older individuals enlisted in nutrition service program in rural northeast Georgia, USA, enrolled between September 1997 and September 1998. | Radioassay. Vitamin B12 deficiency: serum vitamin B12 <258 pmol/L, serum MMA >271 nmol/L, MMA concentration >methylcitric acid concentration) | Method not specified. Anaemia (women Hb<120 g/L and men Hb<130 g/L): prevalence 22% | Unclear:  1) Vitamin B12 deficient subjects twice as likely to be anaemic as the non-deficient subjects: 38% and 18%, respectively.  2) Mean (SD) Hb in vitamin B12 deficient subjects (n=24) 126 (21) g/L; in non-deficient subjects (n=79) 130 (15) g/L, NS.  3) Mean (SD) MCV in vitamin B12 deficient subjects 91.8 (4.5) fL; in non-deficient subjects 90.6 (6.6) fL, NS. | 4 points |  |
| *Based on checklists from van der Windt et al [23,24] | | | | | | | | | |

**Additional file 3** continued

| Author | Year of publication | Sample size (N) | Age of subjects (years) | Study population | Vitamin B12 (VitB12): analysis and limits for deficiency | Haemoglobin (Hb): analysis and limits for anaemia | Presence of an association | Quality of study* |  |
| --- | --- | --- | --- | --- | --- | --- | --- | --- | --- |
| Lippi [33] | 2009 | 878 | Range 85-101 | Unselected subjects older than 85 years, who were referred by general practitioners to a laboratory in Verona, Italy, for routine diagnostic check-up over a period of 2 years | Method not specified. Vitamin B12 deficiency: <203.3 pg/mL | Method not specified. Anaemia: Hb<13.2 g/dL in men and Hb<12.2 g/dL in women. Prevalence of anaemia: 83% in men and 76% in women | No:  1) Vitamin B12 not associated with haemoglobin in a multivariable linear regression analysis (men: r=-0.001, p=0.15, women: r=-0.001, p=0.32).  2) No statistically significant difference in vitamin B12 between anaemic and non-anaemic subjects in both sexes (men mean (SEM) vitamin B12 426.9 (319.9) pg/mL vs 321.2 (119.3) pg/mL [p=0.12], women 444.6 (245.3) pg/mL vs 441.9 (105.7) pg/mL [p=0.07]).  3) Prevalence of vitamin B12 deficiency similar in anaemic and non-anaemic subjects: men 28% vs 36% [p=0.07], women 22% vs 16% [p=0.16] | 6 points |  |
| Loikas [34] | 2007 | 1048 | ≥65, 37% ≥75 | Lieto study; unselected population based health survey in Lieto, Finland. Data collected between March 1998 and December 1999. | Competitive protein binding assay. Vitamin B12 deficiency (B12<150 pmol/L, or increased tHcy (>15µmol/l) and low holoTC (<37 pmol/L)) n=97; No vitamin B12 deficiency (n=924) | Automated haematology analyzer. Anaemia (Hb women Hb <117 g/L, men Hb<134 g/L: prevalence 13% | No:  1) Vitamin B12 deficiency more frequent in subjects with anaemia than in those without anaemia (15% vs 8.6%, p=0.013), but adjustment for age and gender removed the effect: OR 1.3, 95% CI 0.7- 2.3.  2) Vitamin B12 deficiency not associated with macrocytosis (OR 1.2 [95% CI 0.6-2.7]) and macrocytic anaemia (OR 1.2 [95% CI 0.3-5.5]). | 6 points |  |
| McLennan [35] | 1973 | 347 | >65 | Random sample of people >65 years living at home in Kilsyth and Northern Glasgow, UK. | Method using *L. Leichmanii*  Vitamin B12 deficiency: vitamin B12 <140 pg/mL (103 pmol/L), prevalence = 5.4% | Manual cyanmethaemoglobin method. Anaemia (Hb<12 g/dL): prevalence 7.5% in men and 20% in women | Unclear:  Prevalence of vitamin B12 deficiency 9% in anaemic subjects and 5% in non-anaemic subjects. | 5 points |  |
| *Based on checklists from van der Windt et al [23,24]. | | | | | | | | | |

| Author | Year of publication | Sample size (N) | Age of subjects (years) | Study population | Vitamin B12 (VitB12): analysis and limits for deficiency | Haemoglobin (Hb): analysis and limits for anaemia | Presence of an association | Quality of study* |  |
| --- | --- | --- | --- | --- | --- | --- | --- | --- | --- |
| Morris [36] | 2007 | 1459 | ≥60, mean 70 (SEM 0.32) | Non-institutionalized civilian population (NHANES), USA. Data collected from 1999-2002. | Radioassay. Low vitamin B12 status: vitamin B12<148 pmol/L or MMA>210 nmol/L for serum vitamin B12-replete participants with normal creatinine concentrations: prevalence 25% | Haematology flow cytometer. Anaemia (women Hb<12 g/dL and men Hb<13 g/dL): prevalence 4.5% | Yes:  1) 3.2% anaemia in subjects with normal vitamin B12; 8.3% anaemia in subjects with low vitamin B12: adjusted OR 2.7 (95% CI 1.7-4.4)  2) Low vitamin B12 associated with macrocytosis (adjusted OR 1.8 [95% CI 1.02-3.1]) | 5 points |  |
| Penninx [37] | 2000 | 700 | ≥65, mean 77.3 | Physically disabled older women living in the community (Women’s Health and Ageing Study), Baltimore area, USA | Competitive protein binding assays. Vitamin B12 deficiency (serum vitamin B12 level <258 pmol/L, MMA level >271 nmol/L, MMA = 2- methylcitric acid level): prevalence 17.3% | Method not specified. Anaemia was defined as Ht<35 mg/dL. | Unclear:  1) No difference in Ht between subjects with B12 deficiency (39.9 mg/dL (sd 4.7)) and non-deficient subjects (39.9 mg/dL (sd 4.2)).  2) Anaemia more common in vitamin B12 deficient subjects than in non-deficient subjects (17.4% vs. 11%, p=0.05).  3) No difference in between subjects with vitamin B12 deficiency and those without (93.5 fL, SD 7.5 vs 93.9 fL, SD 6.5) | 4 points |  |
| *Hospitalized/Institutionalized* | | | | | | | | | |
| Bisbe [38] | 2009 | 599 | Mean 68 (SD 13) | All consecutive patients scheduled for major orthopaedic surgery for which blood was routinely grouped preoperatively in University Hospital in Barcelona, Spain. Data collected from January 2001 and December 2002. | Radioimmunoanalysis. Vitamin B12 deficiency: vitamin B12 ≤270 pg/mL | Advia cell counter. Anaemia was defined as Hb<12 g/dL for women and Hb<13 g/dL for men. Prevalence: 10.5% | No:  1) No differences in vitamin B12 between anaemic and non-anaemic patients: mean vitamin B12 538 (SD 559) pg/mL vs 523 (SD 393) pg/mL, p>0.05.  2) No difference in prevalence of vitamin B12 deficiency between anaemic and non-anaemic patients (11.3% vs 4.9%, p>0.05) | 5 points |  |
| *Based on checklists from van der Windt et al [23,24] | | | | | | | | | |

**Additional file 3** continued

**Additional file 3** continued

| Author | Year of publication | Sample size (N) | Age of subjects (years) | Study population | Vitamin B12 (VitB12): analysis and limits for deficiency | Haemoglobin (Hb): analysis and limits for anaemia | Presence of an association | Quality of study* |  |
| --- | --- | --- | --- | --- | --- | --- | --- | --- | --- |
| Chui [39] | 2001 | 3453 | 48% >70 | All patients admitted to the Prince of Wales hospital, Hong Kong with vitamin B12 measurements in 1996. | Microparticle enzyme intrinsic factor assay. Definite vitamin B12 deficiency: vitamin B12 level <140 pmol/L: prevalence 6.6% | Method not specified. Anaemia (women Hb<11.5 g/dL and men Hb<13 g/dL). | No:  1) 6.0% of subjects with anaemia and 5.2% of subjects without anaemia had vitamin B12 <140 pmol/L, p<0.2.  2) 9.8% of subjects with macrocytosis and 4.6% of subjects with normocytosis had B12 <140 pmol/L, p<0.001 | 5 points |  |
| Joosten [40] | 1990 | 292 | >65 | Consecutive patients admitted to the geriatric department of the University Hospital, Leuven, Belgium | Radioassay. Low vitamin B12 levels (<110 pmol/L): N=22 (7.5%) | Coulter S plus IV. Anaemia (Hb ≤7.14 mmol/L): N=80 (27%) | Unclear:  1) Median (P5-P95) vitamin B12 for subjects with anaemia 282 (65-1218) pmol/L and for subjects without anaemia 237 (103-1476) pmol/L (Not significant).  2) Median Hb for subjects with vitamin B12<110 pmol/L 7.63 (4.84-8.94) mmol/L and for subjects with vitamin B12≥110 pmol/L 8.07 (5.46-9.81) mmol/L, p=0.04.  3) Median MCV for subjects with vitamin B12<110 pmol/L 95 (82-105) fL and for subjects with vitamin B12≥110 pmol/L 93 (80-104) fL, NS | 5 points |  |
| Kwok [41]† | 2002 | 96 | >55, mean age >78.0 | Female ambulatory vegetarians (>3 years) in Hong Kong. | Solid phase no-boil Dual count radioimmunoassay. Definite vitamin B12 deficiency: vitamin B12<150 pmol/L and MMA>0.4 mmol/L (29.1%). Possible vitamin B12 deficiency: vitamin B12<150 pmol/L or MMA>0.4 mmol/L (46.9%) | Maxim Cell Count analyzer. Anaemia: Hb<12 g/dL. | Unclear:  1) Mean Hb (SD) for normal vitamin B12: 13.0 g/dL (1.3); for possible vitamin B12 deficiency: 12.1 g/dL (1.0); for definite vitamin B12 deficiency: 12.4 g/dL (1.2) p=0.03. After adjustment for confounding variables no association between vitamin B12 and haematological indices.  2) Mean MCV (SD) for normal vitB12 status: 90.6 fL (9.6); for possible vitB12 deficiency: 88.8 fL (8.2); for definite B12 deficiency: 90.2 fL (9.0), NS. | 5 points |  |
| *Based on checklists from van der Windt et al [23,24]  † >51.1% of the study population lived in old age home residence | | | | | | | | | |

**Additional file 3** continued

| Author | Year of publication | Sample size (N) | Age of subjects (years) | Study population | Vitamin B12 (VitB12): analysis and limits for deficiency | Haemoglobin (Hb): analysis and limits for anaemia | Presence of an association | Quality of study* |  |
| --- | --- | --- | --- | --- | --- | --- | --- | --- | --- |
| Metz [42] | 1996 | Cases: 43, Controls: 51 | Cases: mean 79.8  Controls: mean 80.7 | Patients with suspected low vitamin B12 levels based on clinical examination by attending staff in Royal Melbourne and North West hospitals, Australia. If low vitamin B12: case. If normal vitamin B12: control. | Radioassay. Cases: vitamin B12<150 pmol/L  Controls: vitamin B12≥150 pmol/L | Method not specified. Reference range for women 115-160 g/L and for men 130-180 g/L. | No:  1) Mean Hb for cases 124.5 (SD 22.4) g/L and for controls 129.7 (SD 16.2) g/L, student t-test: p=0.251  2) Mean MCV for cases 95.6 (SD 9.2) fL and for controls 93.2 (SD 5.1) fL, student t-test: p=0.127 | 2 points |  |
| Mooney [43]† | 2004 | 905 | 65-85 | Hospitalized patients in Belfast, Ireland, who had vitamin B12, folate, Hb, MCV and ferritin measured within ±4 days of each other in February-July 2003 | Method not specified. Population divided in quintiles of vitamin B12, and stratified as frankly deficient (<200 ng/L [148 pmol/L]), suboptimally deficient (200-249 ng/L [148-184 pmol/L]), possibly deficient (250-349 ng/L [185-257 pmol/L]) and normal (>350 ng/L [258 pmol/l]) | Method not specified. Low Hb: <130 g/L for males and <115 g/L for females | No:  1) No difference in prevalence low Hb between quintiles of vitamin B12 (Q1: 55%, Q2: 53%, Q3: 52%, Q4: 54% and Q5: 63%) or between groups of vitamin B12 status (frankly deficient: 54%, suboptimally deficient: 53%, possibly deficiency: 54%, and normal: 58%).  2) No difference in prevalence high MCV(>100fL) between groups of vitamin B12 status (frankly deficient: 10%, suboptimally deficient: 12%, possibly deficiency: 7%, and normal: 9%). | 3 points |  |
| Prayurahong [44] | 1993 | 147 | ≥60 | Subjects visiting clinic for older individuals in Rajvithi Hospital, Bangkok | Radioimmunoassay. Vitamin B12 deficiency (vitamin B12 <200 pg/mL [148 pmol/L]): prevalence 6.9% (8.8% in women and 3.7%) in men) | Modified cyanmethaemoglobin method. Anaemia (Hb<12 g/dL in women and Hb<13 g/dL in men): prevalence 15% (13% in women and 18.2% in men) | Unclear:  1) No statistically significant correlation between Hb and vitamin B12.  2) Tendency in females for vitamin B12 to decrease as Hb increased. | 3 points |  |
| *Based on checklists from van der Windt et al [23,24]  †Additional information in Cuskelly GJ, Mooney KM, and Young IS: **Folate and vitamin B12: friendly or enemy nutrients for the elderly.** *Proc Nutr Soc* 2007, **66**:548-558. | | | | | | | | | |

**Additional file 3** continued

| Author | Year of publication | Sample size (N) | Age of subjects (years) | Study population | Vitamin B12 (VitB12): analysis and limits for deficiency | Haemoglobin (Hb): analysis and limits for anaemia | Presence of an association | Quality of study* |  |
| --- | --- | --- | --- | --- | --- | --- | --- | --- | --- |
| Stott [45] | 1997 | 290 | Range 62-110 | Consecutive new referrals to a geriatric medical unit in Glasgow, Scotland. | Radiosorbent assay. Low vitamin B12 levels: <175 pmol/L + normal ferritin and folate levels (N=37). Reference group: vitamin B12 >175 pmol/L, ferritin ≥100 ng/ml, erythrocyte folate ≥75 ng/mL and no known chronic inflammatory disease (N=253) | Coulter S plus IV method | No:  1) Mean Hb levels were not significantly reduced in those with low vitamin B12. Men; 140 (sd 12) vs 137 (sd 22) g/L. Women: 128 (sd 10) vs 129 (sd 18) g/L (NS).  2) Those with low vitamin B12 had ~~an~~ elevated MCV compared to control subjects (96.0 [SD 6.7] fL vs 91.7 [SD 6.0], p<0.001). | 5 points |  |
| Wang [46] | 2009 | 827 | Mean 77.1 (SD 7.5), range 60-96 | Patients in the department of Neurology of Shanghai Punan Hospital, Shanghai, China, from March 2007 to July 2008. | Chemiluminescent microparticle immunoassay technology. Vitamin B12 deficiency: vitamin B12 <189 pg/mL and homocysteine concentrations >15μmol/L. Prevalence 19.7% | Method not specified. | Yes:  1) Vitamin B12 deficient patients had lower haemoglobin than patients with normal vitamin B12.  2) Vitamin B12 deficient patients had significantly higher MCV than patients with normal vitamin B12 levels (p<0.001). | 4 points |  |
| Witte [47] | 2004 | 296 | Mean 72.5 (10.3) | Consecutive patients with chronic heart failure attending a heart failure clinic in Cottingham, UK | Method unknown. Low vitamin B12 levels: <180 ng/L (133 pmol/L) | Method not specified. Moderate anaemia (Hb<11.5 g/dL), mild anaemia (Hb 11.6-12.5 g/dL), normal Hb (>12.5 g/dL) | No:  No correlation between vitamin B12 and haemoglobin. | 4 points |  |
| *Based on checklists from van der Windt et al [23,24] | | | | | | | | | |
