# Supplementary material for: Subnormal vitamin B12 concentrations and anaemia in older people: a systematic review
Source: BMC Geriatr. 2010 Jun 23;10:42. doi: 10.1186/1471-2318-10-42 (PMC2900261; doi:10.1186/1471-2318-10-42)
Supplement: Additional file 4 — Quality assessment of cross-sectional observational studies on aetiology of vitamin B12 deficiency and anaemia in elderly subjects included in the present review [file 1471-2318-10-42-S4.DOC]

| Author | Allain [27] | Björkegren [28] | Clarke [29] | Hin [30] | Hvas [31] | Johnson [32] | Lippi [33] |
| --- | --- | --- | --- | --- | --- | --- | --- |
| Year | 1997 | 2001 | 2008 | 2006 | 2005 | 2003 | 2009 |
| Journal | Cent Afr J Med | J Intern Med | Br J Nutr | Age Ageing | J Intern Med | Am J Clin Nutr | Arch Intern Med |
|  |  |  |  |  |  |  |  |
| *Study population* |  |  |  |  |  |  |  |
| Were valid selection criteria used for the study population? | Yes | Yes | Yes; | Yes | No | Yes | Yes |
| Did more than 80% of the eligible subjects participate in the study? | Yes | Yes | No | ? | No | ? | Yes |
|  |  |  |  |  |  |  |  |
| *Exposure assessment* |  |  |  |  |  |  |  |
| Was the exposure measured with a valid and reproducible method? | Yes | Yes | Yes | Yes | Yes | Yes | Yes |
|  |  |  |  |  |  |  |  |
| *Outcome assessment* |  |  |  |  |  |  |  |
| Was the outcome measured with a valid and reproducible method? | Yes | Yes | Yes | Yes | Yes | Yes | Yes |
| Were only new and incident patients used? | No | No | Yes | No | Yes | No | No |
|  |  |  |  |  |  |  |  |
| *Analysis* |  |  |  |  |  |  |  |
| Were the results adjusted for possible confounders? | No | Yes | Yes | No | Yes | No | Yes |
| Were more than 100 subjects included in the study* | Yes | Yes | Yes | Yes | Yes | Yes | Yes |
|  |  |  |  |  |  |  |  |
| Total score | 5 points | 6 points | 6 points | 4 points | 5 points | 4 points | 6 points |
|  |  |  |  |  |  |  |  |
| Based on checklists from van der Windt et al [23,24].  No or ? = 0 points  Yes = 1 point  *more than 50 participants was a requirement for inclusion in the review. Studies with more than 100 participants were rewarded with an additional point. | | | | | | | |

**Additional file 4** Quality assessment of cross-sectional observational studies on aetiology of vitamin B12 deficiency and anaemia in elderly subjects included in the present review

**Additional file 4** continued

| Author | Loikas [34] | McLennan [35] | Morris [36] | Penninx [37] | Bisbe [38] | Chui [39] | Joosten [40] |
| --- | --- | --- | --- | --- | --- | --- | --- |
| Year | 2007 | 1973 | 2007 | 2000 | 2008 | 2001 | 1990 |
| Journal | Age Ageing | Q J Med | Am J Clin Nutr | Am J Psychiatry | Transfus Altern Transfus Med | Nutrition | Ned Tijdschr Geneeskd |
|  |  |  |  |  |  |  |  |
| *Study population* |  |  |  |  |  |  |  |
| Were valid selection criteria used for the study population? | Yes | Yes | Yes | Yes | Yes | Yes | Yes |
| Did more than 80% of the eligible subjects participate in the study? | No | No | ? | No | Yes | Yes | Yes |
|  |  |  |  |  |  |  |  |
| *Exposure assessment* |  |  |  |  |  |  |  |
| Was the exposure measured with a valid and reproducible method? | Yes | Yes | Yes | Yes | Yes | Yes | Yes |
|  |  |  |  |  |  |  |  |
| *Outcome assessment* |  |  |  |  |  |  |  |
| Was the outcome measured with a valid and reproducible method? | Yes | Yes | Yes | Yes | Yes | Yes | Yes |
| Were only new and incident patients used? | Yes | Yes | No | No | No | No | No |
|  |  |  |  |  |  |  |  |
| *Analysis* |  |  |  |  |  |  |  |
| Were the results adjusted for possible confounders? | Yes | No | Yes | No | No | No | No |
| Were more than 100 subjects included in the study* | Yes | Yes | Yes | Yes | Yes | Yes | Yes |
|  |  |  |  |  |  |  |  |
| Total score | 6 points | 5 points | 5 points | 4 points | 5 points | 5 points | 5 points |
|  |  |  |  |  |  |  |  |
| Based on checklists from van der Windt et al [23,24].  No or ? = 0 points  Yes = 1 point  *more than 50 participants was a requirement for inclusion in the review. Studies with more than 100 participants were rewarded with an additional point. | | | | | | | |

**Additional file 4** continued

| Author | Kwok [41] | Metz [42] | Mooney [43]† | Prayurahong [44] | Stott [45] | Wang [46] | Witte [47] |
| --- | --- | --- | --- | --- | --- | --- | --- |
| Year | 2002 | 1996 | 2004 | 1993 | 1997 | 2009 | 2004 |
| Journal | Am J Hematol | J Am Geriatr Soc | Proc Nutr Soc | J Med Assoc Thai | Br J Nutr | Neuroscience Bull | Am Heart J |
|  |  |  |  |  |  |  |  |
| *Study population* |  |  |  |  |  |  |  |
| Were valid selection criteria used for the study population? | Yes | No | ? | ? | Yes | Yes | Yes |
| Did more than 80% of the eligible subjects participate in the study? | ? | ? | ? | ? | Yes | ? | Yes |
|  |  |  |  |  |  |  |  |
| *Exposure assessment* |  |  |  |  |  |  |  |
| Was the exposure measured with a valid and reproducible method? | Yes | Yes | Yes | Yes | Yes | Yes | ? |
|  |  |  |  |  |  |  |  |
| *Outcome assessment* |  |  |  |  |  |  |  |
| Was the outcome measured with a valid and reproducible method? | Yes | Yes | Yes | Yes | Yes | Yes | Yes |
| Were only new and incident patients used? | Yes | ? | ? | ? | No | ? | No |
|  |  |  |  |  |  |  |  |
| *Analysis* |  |  |  |  |  |  |  |
| Were the results adjusted for possible confounders? | Yes | No | No | No | No | No | No |
| Were more than 100 subjects included in the study* | No | No | Yes | Yes | Yes | Yes | Yes |
|  |  |  |  |  |  |  |  |
| Total score | 5 points | 2 points | 3 points | 3 points | 5 points | 4 points | 4 points |
|  |  |  |  |  |  |  |  |
| Based on checklists from van der Windt et al [23,24].  No or ? = 0 points  Yes = 1 point  *more than 50 participants was a requirement for inclusion in the review. Studies with more than 100 participants were rewarded with an additional point.  †Additional information in Cuskelly GJ, Mooney KM, and Young IS: **Folate and vitamin B12: friendly or enemy nutrients for the elderly.** *Proc Nutr Soc* 2007, **66**:548-558. | | | | | | | |
