# Supplementary material for: Subnormal vitamin B12 concentrations and anaemia in older people: a systematic review
Source: BMC Geriatr. 2010 Jun 23;10:42. doi: 10.1186/1471-2318-10-42 (PMC2900261; doi:10.1186/1471-2318-10-42)
Supplement: Additional file 5 — Observational longitudinal study on aetiology of vitamin B12 deficiency and anaemia in older subjects included in the present review [file 1471-2318-10-42-S5.DOC]

**Additional file 5** Observational longitudinal study on aetiology of vitamin B12 deficiency and anaemia in older subjects included in the present review

| Author | Year of publication | Sample size (N) | Age of subjects (years) | Study population | Vitamin B12 (VitB12): analysis and limits for deficiency | Haemoglobin (Hb): analysis and limits for anaemia | Presence of an association | Quality of study*† |  |
| --- | --- | --- | --- | --- | --- | --- | --- | --- | --- |
| Den Elzen [48] | 2008 | 423 | 85 | All 85-year-old inhabitants of Leiden, the Netherlands. Participants using vitamin B12 or folate supplements at baseline or during follow-up were excluded. Participants were enrolled between September 1997 and September 1999. | Dual Count Solid Phase No Boil Assay. Vitamin B12 deficiency: <150 pmol/L: prevalence 16.1% | Automated analysis system. Anaemia: Hb<12 g/dL for women and Hb<13 g/dL for men. | No:  1) Vitamin B12 deficiency was not associated with the presence of anaemia at baseline (adjusted OR 1.51 95% CI 0.79-2.87), nor with developing anaemia during follow-up (adjusted HR 0.92 95% CI 0.46-1.82).  2) At baseline, median MCV was higher in those with low vitamin B12 than in those with normal vitamin B12 (93 fL (IQR 90-96) and 91 fL (IQR 88-94), respectively). There were no differences in the change~~s~~ in MCV during follow-up (p=0.77). | 7 points |  |
| *Based on checklists from van der Windt et al [23,24]  †Additional independent assessment by DAWM van der Windt | | | | | | | | | |
