# Supplementary material for: Subnormal vitamin B12 concentrations and anaemia in older people: a systematic review
Source: BMC Geriatr. 2010 Jun 23;10:42. doi: 10.1186/1471-2318-10-42 (PMC2900261; doi:10.1186/1471-2318-10-42)
Supplement: Additional file 6 — Quality assessment of longitudinal observational studies on aetiology of vitamin B12 deficiency and anaemia in elderly subjects included in the present review [file 1471-2318-10-42-S6.DOC]

**Additional file 6** Quality assessment of longitudinal observational studies on aetiology of vitamin B12 deficiency and anaemia in elderly subjects included in the present review

| Author | Den Elzen [48] |
| --- | --- |
| Year | 2008 |
| Journal | Arch Intern Med |
|  |  |
| *Study population* |  |
| Were valid selection criteria used for the study population? | Yes |
| Did more than 80% of the eligible subjects participate in the study? | Yes |
| Was the response at main moment of follow up >80 | ? |
|  |  |
| *Exposure assessment* |  |
| Was the exposure measured with a valid and reproducible method? | Yes |
|  |  |
| *Outcome assessment* |  |
| Was the outcome measured with a valid and reproducible method? | Yes |
| Were only new and incident patients used? | ? |
| Were data collected for ≥1 year? | Yes |
|  |  |
| *Analysis* |  |
| Were the results adjusted for possible confounders? | Yes |
| Were more than 100 subjects included in the study* | Yes |
|  |  |
| Total score | 7 points |
|  |  |
| Based on checklists from van der Windt et al [23,24]  No or ? = 0 points  Yes = 1 point  *more than 50 participants was a requirement for inclusion in the review. Studies with more than 100 participants were rewarded with an additional point. | |
