# Supplementary material for: Subnormal vitamin B12 concentrations and anaemia in older people: a systematic review
Source: BMC Geriatr. 2010 Jun 23;10:42. doi: 10.1186/1471-2318-10-42 (PMC2900261; doi:10.1186/1471-2318-10-42)
Supplement: Additional file 7 — Randomized placebo-controlled trials of the effect of vitamin B12 administration on haemoglobin levels in older subjects included in the present review [file 1471-2318-10-42-S7.DOC]

**Additional file 7** Randomized placebo-controlled trials of the effect of vitamin B12 administration on haemoglobin levels in older subjects included in the present review

| Author | Year of publication | Sample size (N) | Age (years) | Study population | Intervention | Outcome measure | Treatment effectiveness | Quality* |  |
| --- | --- | --- | --- | --- | --- | --- | --- | --- | --- |
| Hughes [49] | 1970 | Placebo n=19, Treatment n=20 | ≥ 65 | Random sample of subjects aged ≥65 years from general practices in a town in Wales, UK. Subjects with vitamin B12 level <150 pg/mL were invited to the trial. None had anaemia or evidence of vitamin B12 neuropathy and none was taking drugs that might interfere with vitamin B12 assays or anticonvulsants that might reduce the serum vitamin B12 level. | Intramuscular hydroxocobalamin (1000μg), twice in the first week, and then at weekly intervals for a further four weeks or placebo containing a matching solution of phenol red (phenylsulphonphtalein 0.075%) | Between-group differences in change in Hb in 5 weeks from baseline. | No:  On average, a small decrease in Hb level but the difference between the mean change in both groups was small and not statistically significant (0.01 +/-0.35 g) | 4 points |  |
| Hvas [50] | 2001 | Placebo n=70, treatment n=70 | Treatment group: Median 75 (range 19-92)  Placebo group: 74 (range 33-88) | 140 subjects in Aarhus, Denmark, with elevated methylmalonic acid (P-MMA, 0.40-2.00 μmol/L) which had not received prior vitamin B12 treatment. Participants were enrolled between November 1998 and March 2000. Exclusion criteria: low Hb levels, low ferritin levels, TSH≥4.1 mIU/L, high creatinine levels, life-threatening disease, treatment with anticoagulants, tropical atoxic neuropathy. | Weekly intramuscular injections of 1 mg cyanocobalamin (n=70) or placebo containing 1 mL of isotonic sodium chloride (n=70) for 1 month | Percentage change in Hb levels after 3 months. | No:  1) Hb change (%) in treatment group 1% and in placebo group 1%, p=0.95 (Student t-test).  2) MCV change (%) in treatment group 0% and in placebo group 0%,  p=0.70 (Student t-test). | 4 points |  |
| Seal [51] | 2002 | Placebo n=11, oral vitamin B12 10 μg n=10, oral vitamin B12 50 μg, n=10 | Mean age placebo 78, vitamin B12 10 μg 82, vitamin B12 50 μg 85 | 31 patients in two geriatric hospitals in Melbourne, Australia with subnormal serum vitamin B12 (100-150 pmol/L) discovered as part of their clinical assessment. Exclusion criteria: known neoplasm, life-threatening or terminal illness, history of malabsorption, pernicious anaemia, anaemia of other cause, prior vitamin B12 treatment or vitamin supplementation, neurological disorder other than stroke. | Placebo (Australian Pharmaceutical Formulary (APF) red mixture and APF hydrobenzoate compound), 10 μg oral cyanocobalamin or 50 μg oral cyanocobalamin daily for 4 weeks | Difference in change in Hb from baseline after 4 weeks (range 27-39 days) | No:  1) Mean change in Hb in g/L (SD) from baseline:  Placebo: 2.3 (9.3), 10 μg: 3.3 (9.8), 50 μg: 0.7 (13.5)  One-way ANOVA p=0.875  2) Mean change in MCV in fL (SD) from baseline:  Placebo: 0.5 (1.3), 10 μg: 1.4 (2.2), 50 μg: 0.6 (1.1)  One-way ANOVA p=0.421 | 2 points |  |
| *Based on checklist from Jadad et al.[25] | | | | | | | | | |
