# Supplementary material for: Subnormal vitamin B12 concentrations and anaemia in older people: a systematic review
Source: BMC Geriatr. 2010 Jun 23;10:42. doi: 10.1186/1471-2318-10-42 (PMC2900261; doi:10.1186/1471-2318-10-42)
Supplement: Additional file 8 — Quality assessment of randomized placebo-controlled trials on the effect of vitamin B12 administration on haemoglobin levels in elderly subjects included in the present review [file 1471-2318-10-42-S8.DOC]

**Additional file 8** Quality assessment of randomized placebo-controlled trials on the effect of vitamin B12 administration on haemoglobin levels in elderly subjects included in the present review

| Author | Hughes [49] | Hvas [50] | Seal [51] |
| --- | --- | --- | --- |
| Year | 1970 | 2001 | 2002 |
| Journal | BMJ | Clin Chem | J Am Geriatr Soc |
| 1) Was the study described as randomized? | Yes; 1 point | Yes; 1 point | Yes; 1 point |
| 2) Was the study described as double blind? | Yes; 1 point | Yes; 1 point | Yes; 1 point |
| 3) Was there a description of withdrawals and dropouts? | Yes; 1 point | No; 0 points | No; 0 points |
| Subtotal | 3 points | 2 points | 2 points |
|  |  |  |  |
| Add 1 point if |  |  |  |
| the method to generate the sequence of randomization was described and it was appropriate | No; 0 points | Yes; 1 point | No; 0 points |
| *Or* |  |  |  |
| the method of double blinding was described and it was appropriate. | Yes; 1 point | Yes; 1 point | No; 0 points |
|  | 1 point | 2 points | 0 points |
| Deduct 1 point if |  |  |  |
| the method to generate the sequence of randomization was described and it was inappropriate | No; 0 points | No; 0 points | No; 0 points |
| *Or* |  |  |  |
| the method of double blinding was described and it was inappropriate. | No; 0 points | No; 0 points | No; 0 points |
|  | 0 points | 0 points | 0 points |
|  |  |  |  |
| Total score | 4 points | 4 points | 2 points |
|  |  |  |  |
| Based on checklist from Jadad et al [25]. | | | |
